# Shift work disorder and its related factors among health-care workers in a Tertiary Care Hospital in Bangalore, India

**DOI:** 10.12669/pjms.345.16026

**Published:** 2018

**Authors:** Jesly Joseph Mathew, Merlyn Joseph, Madonna Britto, Bobby Joseph

**Affiliations:** 1 Ms. Jesly Joseph Mathew, Post Graduate Student, St. John’s College of Nursing, Bangalore, India; 2 Dr. Merlyn Joseph, Tutor, Department of Community Health, St. John’s Medical College Hospital, Bangalore, India; 3 Prof. Madonna Britto, Department of Medical Surgical Nursing, St. John’s College of Nursing, Bangalore, India; 4 Prof. Bobby Joseph, Department of Community Health, St. John’s Medical College Hospital, Bangalore, India

**Keywords:** Excessive day time sleepiness, Hospital worker, Insomnia, Shift work disorder

## Abstract

**Background & Objective:**

Shift work disorder is the presence of excessive day time sleepiness and insomnia affecting people whose work hours overlap with the typical sleep period. Shift work has pronounced effect on physical and mental health of an individual. Our objective was to estimate the prevalence of symptoms of shift work disorder and the factors associated with it among hospital staff in a tertiary care hospital in Bangalore, India.

**Methods:**

This cross-sectional study was conducted at a private teaching tertiary hospital among 318 hospital workers in Bangalore during September to December 2015. Stratified random sampling method was used and the study population was divided into five strata based upon their parent department. Socioeconomic details, work profile and standard tools– Insomnia severity index, The Epworth sleepiness scale and the FACIT fatigue scale were used.

**Results:**

Of the 318 workers, 290 (91.2%) were females, between 21-25 years of age. Most had work experience of ≤4 years (77.6%). Insomnia was reported among 39.9%, and fatigue among 4.7% of hospital staff. Around 19.8% staff had excessive daytime sleepiness out of which 2.8% warranted medical attention.

**Conclusion:**

Periodic screening during annual medical check-ups and health education on practicing good sleep hygiene will help address this important issue of shift work disorders among workers.

## INTRODUCTION

Shift work is commonly regarded as work that extends outside the daylight hours. “Shift work” commonly denotes those work schedules that divide 24 hours in a day into roughly equal sized time schedules which will be covered by different teams in sequence. The teams alternate between early morning, afternoon, and night shifts or may work a permanent shift.^1^ The past few decades have witnessed a tremendous growth in the population of shift workers, especially in the industrial sector.

Work hours that displace sleep to the daytime and work to the night time interferes with the circadian and homeostatic regulation of sleep. In about 5 to 10 percent of night-shift workers, the sleep-wake disturbance is severe enough to warrant diagnosis as shift-work sleep disorder, which is characterized by a level of excessive sleepiness during night work and clinically significant insomnia during daytime.^2^ Such work hours will in several ways constitute a health problem with respect to sleep and fatigue; cardiovascular disease, accidents, and cancer.^3^ All these symptoms can be attributed to the fact that shift workers experience an out of phase sleep wake cycle which is in direct conflict with their biological circadian rhythm.^4^

It has been documented that people with shift work sleep disorder not only miss family functions and social gatherings but also suffer from a myriad of health problems such as ulcers, sleepiness-related accidents, absenteeism, and depression.^4^

The health-care sector is one prime example wherein people are employed in round the clock shifts. However, there is widespread ignorance and negligence on the part of the health authorities and medical staff when it comes to addressing the health problems posed by shift work.^5^ The present study was undertaken to assess the prevalence of shift work disorder among health-care workers and the likely risk factors associated with it. This study will help us to plan interventions in the future to address this important issue. This would go a long way in improving the overall quality of life of the health-care workers as well as their work performance and satisfaction.

In this study we had the following objectives:

1. To estimate the prevalence of symptoms of shift work disorder among hospital staff in a tertiary care hospital in Bangalore.
2. To determine the factors associated with shift work disorder among hospital staff in a tertiary care hospital in Bangalore.

## METHODS

This cross-sectional study was conducted at a private teaching tertiary hospital located in Bangalore during September to December 2015. Hospital workers belonging to the following departments or occupations – nursing, housekeeping, hospital aide, nursing aide and laboratory technicians were our study population. Workers aged <60 years who had a minimum of seven days of night duty in the last one month were our study subjects. Those workers who were on any medication for neurological and sleep disorders were excluded.

A pilot study was conducted among 35 hospital staff to ascertain the feasibility and ease of administering the questionnaire, the availability and cooperation from the hospital staff and to determine the method of statistical analysis. The sample size was calculated to be 318. Ethical clearance was obtained from the Institution Ethics Committee, St. John’s National Academy of Health Sciences, Bangalore, India.

Stratified random sampling method was used to recruit the study participants. The study population was divided into five strata based upon their parent department. A total of 318 hospital staff took part in this study which included 190 nurses, 51 nursing aides, 32 hospital aides, 25 housekeeping staff and 20 laboratory technicians. A list of all workers in these five departments was obtained from the HR department of the hospital. Computer generated random numbers were used to recruit our study subjects. Informed written consent was obtained and structured interview schedule and standardized questionnaires were used, which are described below.

### Structured interview schedule

The interview schedule consisted of two parts. The first part included the socio-demographic details such as age, gender, place of residence, marital status, type of family, area of work, years of experience, working hours, and frequency of night shifts per month among other details.

Second part of the interview schedule consisted of questions to assess the presence of symptoms of shift work disorder. Standardized tools namely the Insomnia severity index, The Epsworth sleepiness scale and the FACIT fatigue scale were used in this study. The method of administration of these tools and their interpretation is available in publications elsewhere.^5^-^8^

## RESULTS

Of the 318 workers 290 (91.2%) were females. Majority were in the age group between 21-25 years.

The socio-demographic characteristics of the study population are shown in Table-I. A majority of the study population resided in hostels (58.8%), was unmarried (68.6%) and belonged to a nuclear family (78.9%). Walking was the most common mode of travel to work (68.6%) and a majority of them resided within two km radius of the hospital.

**Table-I:** Socio-demographic characteristics (n=318).

S. No.	Baseline variable	Frequency	Percentage (%)
1	*Age (in years)*		
	16-20	23	7.2
	21-25	174	54.7
	26-30	82	25.8
	31-35	18	5.7
	36 and above	21	6.6
2	*Gender*		
	Male	28	8.8
	Female	290	91.2
3	*Residing at*		
	Hostel	187	58.8
	Home	131	41.2
4	*Marital status*		
	Single	218	68.6
	Married	100	31.4
5	*Type of family*		
	Nuclear	249	78.9
	Joint	69	21.1
6	*No. of family members*		
	0-2	240	7.5
	3-5	205	64.5
	>5	89	28
7	*Distance from workplace*		
	0-2km	220	69.2
	2-4km	23	7.2
	4-6km	24	7.6
	>6km	51	16
8	*Mode of transport*		
	Walking	218	68.6
	Public Bus	85	26.7
	2 wheeler	11	3.5
	Hired 3 wheeler	4	1.3

The description of the nature of work is shown in Table-II. Majority of the study subjects worked in general wards (36.2%), commonly in 6 hour shifts (56.3%) and had night duties at least twice a month (58.8%). Majority of them worked for 12 continuous hours (66.03%) without rest and majority of them had a work experience of less than or equal to 4 years (77.6%).

**Table-II:** Description of the nature of work (n=318).

S. No	Variable	Frequency	Percentage (%)
1	*Designated department*		
	General wards	115	36.2
	Private wards	68	21.4
	Intensive Treatment Units	29	9.1
	Intensive Care Units	49	15.4
2	*Working hours*		
	6 hours	179	56.3
	8 hours	139	43.7
3	*Frequency of night duty per month*		
	Once	131	41.2
	Twice	187	58.8
4	*Duration of night duty per day*		
	12 hours without rest	210	66.03
	12 hours with 2 hours of rest	108	33.97
5	*Years of experience*		
	≤4 years	247	77.6
	>4 years	71	22.3

Insomnia was reported among 39.9% of the staff (Table-III). A majority of the workers had no symptoms of insomnia (60.1%), clinical insomnia was noted among 4.7% of the workers.

**Table-III:** Prevalence of symptoms of insomnia using Insomnia severity index (n=318).

Presence of insomnia	Frequency	Percentage (%)
No clinically significant insomnia	191	60.1
Sub threshold insomnia	112	35.2
Clinical insomnia moderate	9	2.8
Clinical insomnia severe	6	1.9

The FACIT Fatigue Scale was used to ascertain fatigue among the workers. Fatigue was reported among 4.7% of the hospital staff, most commonly among nursing aides (19.6%). There was a significant association (p<0.05) between duration of working hours (>8 hours), frequency of night duty per month (twice per month) and duration of night duty (12 hours without rest) with reports of fatigue.

Around 19.8% of study subjects had excessive daytime sleepiness out of which 2.8% warranted medical attention.

Additionally, there was no association between number of family members, part time job and excessive daytime sleepiness.

Additionally, there was no significant association between place of residence, marital status, and type of family, number of children, distance from work place and mode of transport with symptoms of insomnia.

## DISCUSSION

The present study was conducted among 318 hospital staff of which the majority belonged to the age group of 21-25 years. This indicates the relatively younger age group of population was exposed to shift work in this tertiary hospital. Majority of the hospital staff were female (91.2%). This could be due to the fact that a large proportion of our study subjects were chosen from the nursing strata (190 subjects), which is still considered to be a predominantly female dominant profession.

**Table-IV:** Prevalence of excessive daytime sleepiness among hospital staff (n= 318).

Epsworth sleepiness scale interpretation	Frequency	Percentage (%)
Unlikely to be abnormally sleepy during daytime	207	65.1
Average amount of daytime sleepiness	48	15.1
Excessively sleepy depending upon the situation. May need medical attention	54	17
Excessively daytime sleepiness, should consider seeking medical attention	9	2.8

Results from the present study showed that 39.9% of hospital staff suffered from shift work disorder. This figure closely correlated with another survey conducted in the United States of America among 110,422 sample adults in the National Health Interview Survey, 2004-2007 who worked in eight industries with high prevalence of shift work disorder. The findings of that survey ranked the health-care industry overall third in the prevalence of insomnia and short sleep duration (32%).^9^

**Table-V:** Association between baseline variables and symptoms of excessive day time sleepiness

Variable	Excessive day time sleepiness	No symptoms	Odds ratio	Chi square value	P value
*Gender*					
Male	5 (17.9%)	23(82.1%)			
Female	106 (36.6%)	184(63.4%)	0.38	3.93	<0.05^*^
*Age*					
≤25	77(39.1%)	120(60.9%)			
>25	34(28.1%)	87(71.9%)	1.64	3.98	<0.05^*^
*Marital status*					
Single	88(40.4%)	130(59.6%)			
Married	23(23%)	77(77%)	2.27	9.10	<0.01^*^
*Type of family*					
Nuclear	94(37.8%)	155(62.2%)			
Joint	17(24.6%)	52(75.4%)	1.86	4.09	<0.05^*^
*Place of residence*					
Hostel	85(45.5%)	102(54.5%)			
Home	26(19.8%)	105(80.2%)	3.37	22.23	<0.001^*^
*Distance from workplace*					
≤4 km	96(39.5%)	147(60.5%)			
>4 km	15(20%)	60(80%)	0.38	9.60	<0.01^*^
*Mode of transport*					
Walking	88(40.4%)	130(59.6%)			
Vehicular	23(23%)	77(77%)	2.27	9.10	<0.01^*^
*Working hours during day duty*					
6 hours	84(46.9%)	95(53.1%)			
8 hours	27(19.4%)	112(80.6)	3.67	26.05	<0.001^*^
*No. of children (n=100)*					
≤2	21(23.6%)	68(76.4%)			
>2	2(18.2%)	9(81.8%)	1.39	0.16	>0.05
*Years of experience*					
≤4 years	93(37.7%)	154(62.3%)			
>4 years	18(25.4%)	53(74.6%)	1.78	3.67	>0.05

In the present study 1.9% of the hospital staff had severe insomnia. Nursing aides accounted for 3.9% of the cases followed by nursing staff (2.9%). Similar studies done among nurses in other locations revealed that shift work does significantly influence sleep quality.^10^,^11^ We found that health-care workers who had >4 years of work experience had greater insomnia as compared to the others. The reason could be due to the fact that those who were exposed to prolonged duration of shift work had other risk factors as well for shift work disorder such as higher levels of responsibility and greater demand at work. Our findings differed from other studies which showed that increase in years of practice was associated with lower symptoms of shift work disorder.^12^ This suggests that the adaptation to night shift work which is usually expected occurs rarely, since usual daytime activity and night-time sleep are resumed on the off duty days and day shift days.

**Table-VI:** Association between baseline variables and insomnia

Variable	Presence of insomnia	No symptoms	Odds ratio	Chi square value	P value
*Gender*					
Male	8(28.6%)	20(71.4)	0.57	1.65	>0.05
Female	119(41%)	171(59%)			
*Age in years*					
≤25	77(39.1%)	120(60.9%)	0.91	0.16	>0.05
>25	50(41.3%)	71(58.7%)			
*Years of experience*					
≤4 year	89(36%)	158(64%)	0.49	7.03	<0.01^*^
>4 year	38(53.5%)	33(46.5%)			
*Working hours during day duty*					
6 hours	80(44.7%)	99(55.3%)	1.58	3.86	<0.05^*^
8 hours	47(33.8%)	92(66.2%)			

Our study also showed that those who worked in shifts of longer duration (8 hours) had decreased insomnia as compared to those who worked for six hours or lesser. This probably might be because in a six hour shift the work is more intensive and stressful wherein the worker has less time to complete the same amount of work as compared to those in longer shifts. They also do not get the privilege of napping in between work hours and hence were more likely to be sleep deprived.

While studies have shown greater prevalence of insomnia in women due to hormonal changes and greater sleep disturbances, no such association was found in this study. Additionally, there was no significant association between place of residence, marital status, and type of family, number of children, distance from work place and mode of transport with symptoms of insomnia.

In the present study 19.8% of study subjects had excessive daytime sleepiness out of which 2.8% warranted medical attention. This is a particularly important finding given the nature of work at the hospital. Errors and accidents involving health-care workers not only can directly impact their own lives (e.g. needle stick injuries) but also put the lives of patients in great jeopardy.

Day time somnolence was observed more among the female workers when compared to the males. Similar findings have been observed in other study settings as well.^13^,^14^ Greater responsibilities at home including child care, could lead to more sleep deprivation in women hence contributing to excessive day time sleepiness. Presence of other secondary sleep disorders and hormonal changes could also attribute to such a finding. Excessive daytime sleepiness was associated with age (≤25 years) and unmarried individuals. Marriage was shown to be a protective factor among males in a similar study done in Japan to assess gender differences in prevalence and risk factors of EDS.^14^ Younger workers with less work experience probably did not have the necessary coping skills and adaptation to shift work as compared to their older and experienced colleagues hence experiencing more daytime sleepiness. This finding could also be due to the ‘healthy worker effect’ wherein the older workers experiencing symptoms of excessive daytime sleepiness have stopped working in shifts altogether thereby leaving a more healthy survivor population in this study. While it is expected that those living in large families and having greater number of children experience more sleep deprivation and interruptions in their sleep pattern, no association was found between them and excessive day time sleepiness (EDS). Also, we surprisingly found no association between work experience and working in other part time jobs with EDS.

In a study conducted among 510 health-care workers in Saudi Arabia using the Pittsburgh Sleep Quality Index (PSQI) and the Epworth Sleepiness Scale (ESS) it was reported that the PSQI global score (*p*<0.001) and the total ESS score (*p*=0.003) were significantly higher in shift work health care professionals.^15^ In another study conducted among 501 hospital employees in Spain to ascertain their sleep habits and excessive daytime sleepiness (EDS), it was reported that 6.6% of hospital workers had excessive daytime sleepiness.^16^

In the present study fatigue was reported among 4.7% of the hospital staff, most commonly among nursing aides (19.6%). Fatigue has been previously linked to shift work not necessarily shift work sleep disorder per se.^1^ There was a significant association (p<0.05) between duration of working hours (>8 hours), frequency of night duty per month (twice per month) and duration of night duty (12 hours without rest) with reports of fatigue. Earlier studies also recommend to restricting the duration of work hours to 8 hours rather than 12 hours.^17^ Such dose-response relationship is important as it indicates an upper limit of night duties that needs to be scheduled for the worker. Fatigue that result from long working hours and shift work not only is a concern for patient safety but also can lead to greater health-care worker burnout, job dissatisfaction and absenteeism. Fatigue due to shift work might be because the worker did not get adequate sleep at home during the daytime due to frequent sleep interruptions by family and social causes.

### Recommendations

Shift work significantly affects both the professional and personal lives of health-care workers. While health-care workers cannot be completely exempted from shift duties, certain changes will improve their quality of life. Behavioral changes such as practicing good sleep hygiene, scheduled napping during shifts and scheduling forward rotating shifts i.e. from morning-afternoon-night shifts are all effective coping strategies. Also, periodic screening for shift work disorder during routine annual medical check-ups and reinforcement and education of the staff for practicing good sleep hygiene will prove beneficial in the long run. Restricting shift work to twice a month or lesser and reducing the duration of shifts to 8 hours are other key preventive strategies.

### Author’s Contributions

**JJM:** Did data collection, data entry and analysis.

**MJ:** Did data analysis and manuscript writing.

**MB and BJ:** Conceived and designed the study and edited the manuscript.

**MJ** takes the responsibility and is accountable for all aspects of the work in ensuring that questions related to the accuracy or integrity of any part of the work are appropriately investigated and resolved.
